# Isoimperatorin alleviates lipopolysaccharide-induced periodontitis by downregulating ERK1/2 and NF-κB pathways

**DOI:** 10.1515/biol-2022-0541

**Published:** 2023-01-24

**Authors:** Lili Fan, Zhenqiang Li, Linlin Gao, Nan Zhang, Wenxiao Chang

**Affiliations:** Department of Stomatology, Shanxi Bethune Hospital, Shanxi Academy of Medical Sciences, Third Hospital of Shanxi Medical University, No. 99, Longcheng Street, Taiyuan, Shanxi Province, 030032, China

**Keywords:** chronic periodontitis, isoimperatorin, oxidative stress, inflammatory, ERK1/2 and NF-κB pathways

## Abstract

Chronic periodontitis is an inflammatory disease characterized by inflammation of the soft tissues of the gums. To combat this disease, more effective drugs are still needed to identify and develop. Isoimperatorin is a kind of a natural compound, which has anti-inflammatory, analgesic, antitumor, antivirus, and other pharmacological effects. However, its possible effects on the progression of chronic periodontitis are still unclear. In this study, we used human periodontal membrane fibroblasts (hPDLCs), human bone marrow-derived macrophages, and found that isoimperatorin reduced hPDLCs viability. In addition, isoimperatorin alleviated the oxidative stress of periodontal membrane cells. Isoimperatorin reduced proinflammatory factor secretion and receptor activator for nuclear factor-κB ligand–induced osteoclast differentiation in periodontal membrane cells. Further, isoimperatorin inhibited the activation of ERK1/2 and nuclear factor-κB pathways. We, therefore, thought isoimperatorin could serve as a promising drug for the treatment of this disease.

## 1 Introduction

Chronic periodontitis is an inflammatory disease characterized by inflammation of the soft tissues of the gums with attachment defects of periodontal ligaments [1]. It is mainly caused by plaque biofilms that destroy the supporting structures of the teeth, resulting in persistent alveolar bone loss and periodontal connective tissue damage [2,3]. Both osteoblasts and osteoclasts are osteocytes, and osteoblasts promote bone formation, while osteoclasts cause bone resorption [4]. Alveolar bone loss is associated with long-term osteoclast activation [5]. Stimulation of resorption of the alveolar bone eventually leads to tooth loss [6]. To combat this disease, more effective drugs are still needed to identify and develop.

Isoimperatorin is a kind of naturally occurring coumarin compound and is one of the active components in *coumarin*, *Angelica dahurica*, *Radix qiansheng*, and *Radix Aristophanae* [7]. Previous studies have found that isoimperatorin has anti-inflammatory, analgesic, antitumor, antivirus, and other pharmacological effects. For example, it could induce apoptosis of carcinoma cells through mediating MAPK/ERK1/2 signaling pathway [8]. Regulating peroxisome proliferators-activated receptor (PPAR) γ and C/EBP α through Akt signaling pathway promotes adipocyte differentiation and prevents diabetes [9]. It can further improve mitochondrial function and protect against acute liver injury caused by carbon tetrachloride. [10,11,12]. By downregulating mammalian target of rapamycin C1 (mTORC1) signaling pathway to activate autophagy, osteoarthritis mice can improve cartilage degeneration [13]. To date, whether isoimperatorin has a therapeutic effect on periodontitis has not been reported. Therefore, the purpose of this study was to test the effects of different concentrations of isoimperatorin on lipopolysaccharide (LPS)-induced periodontal membrane cell viability, oxidative stress, inflammatory factor secretion, and receptor activator for nuclear factor-κB ligand (RANKL)-induced osteoclast differentiation, and to explore its mechanism.

In this study, we used human periodontal membrane fibroblasts (hPDLCs), human bone marrow-derived macrophages, and found that isoimperatorin reduced LPS-induced periodontal cell viability, alleviated oxidative stress of periodontal cells, reduced secretion of pro-inflammatory factors, and reduced osteoclast formation. Further studies demonstrated that isoimperatorin inhibited LPS-induced ERK1/2 and nuclear factor (NF)-κB pathway activation in periodontal cells. We therefore believed that isoimperatorin could serve as a promising drug for the treatment of this disease.

## 2 Materials and methods

### 2.1 Cell culture

hPDLCs were purchased from the BeNa Culture Collection (Beijing, China) and maintained in Dulbeccos modified eagle medium/F12 medium with 10% fetal bovine serum and 100 μg/mL streptomycin and 100 U/mL penicillin at 37°C with 5% CO_2_. Isoimperatorin was bought from sigma (CAS-no: 482-45-1, USA). hPDLCs were stimulated with isoimperatorin at 0, 5, 10, 25, 50, and 100 μM. LPS (5 μg/mL) was used to induce inflammation in hPDLCs.

### 2.2 Cell viability

The immunocompromised hPDLC cells after indicated treatment were seeded into 96-well plates at the density of 1 × 10^3^ cells/well. Cell viability was detected with CCK-8 kit (Bimake, Houston, USA). Briefly, cells were plated into 96-well plates at about 10^4^ cells/well and treated with CCK-8 solution for 2 h. The absorbance was detected with a microplate reader at 450 nm wavelength.

### 2.3 TUNEL staining

Cells were fixed with 4% formaldehyde, rinsed with phosphate-buffered saline (PBS), and then stained with a cell death detection kit (Roche Molecular Biochemicals, Mannheim, Germany) according to the manufacturer’s protocol. The cells were examined using a microscope (Olympus), and images were taken. The apoptotic cells were counted manually.

### 2.4 Superoxide dismutase (SOD), malondialdehyde (MDA), glutathione, r-glutamyl cysteingl + glycine (GSH), and myeloperoxidase (MPO) detection

After indicated treatment on hPDLCs, cells were collected for detection of MDA, SOD, GSH, and MPO with relevant commercial kits according to manufacturer’s instructions (Nanjing Jiancheng Bioengineering Institute).

### 2.5 Enzyme-linked immunosorbent assay (ELISA)

After indicated stimulations, cell supernatants were collected and used for ELISA assay to detect the level of tumor necrosis factor (TNF)-α, interleukin (IL)-6, IL-18, monocyte chemoattractant protein (MCP)-1, and IL-1β following the manufacturer’s guidelines. The ELISA kits were obtained from Shanghai Xitang Biotechnology Co., Ltd. (Shanghai, China).

### 2.6 Tartrate-resistant acid phosphatase (TRAP) staining

TRAP staining represents the gold standard for the characterization of osteoclasts. Osteoclasts are characterized by their expression of TRAP. Cells were fixed with 4% formalin for 10 min, permeabilized with PBS containing 0.1% Triton X-100 for 10 min, and incubated for 10 min with a TRAP-staining solution (Sigma-Aldrich, St. Louis, MO, USA).

### 2.7 Western blotting

Proteins were extracted with RIPA buffer (Beyotime). Then, the samples were collected, electrophoresed by 10% sodium dodecyl sulphate-polyacrylamide gel electrophoresis, transferred onto polyvinylidene difluoride membranes, and then blocked with 5% fat-free milk in tris-buffered saline and tween 20 buffer. Subsequently, membranes were incubated with primary antibodies targeting p-p65 (1:1,000, Abcam, Cambridge, UK), p65 (1:1,000, Abcam), p-IκBα (1:1,000, Abcam), IκBα (1:1,000, Abcam), ERK1/2 (1:1,000, Abcam), Cleaved-caspase3 (1:1,000, Abcam), p-ERK1/2 (1:1,000, Abcam), and β-actin (1:10,000, Abcam) for 1 h at room temperature. Ultimately, the membranes were conjugated with specific secondary antibodies at room temperature for 1 h.

### 2.8 Statistics

Statistical analysis was performed with GraphPad 6.0. One-way analysis of variance and student’s *t-*test were used for statistical comparisons. All data were presented as mean ± standard error of mean. Three replicates were performed for each experiment. * indicates *p* < 0.05 and significance. ***, *p* < 0.001 vs control, ^, *p* < 0.05, ^^, *p* < 0.01, ^^^, *p* < 0.001 vs LPS.

## 3 Results

### 3.1 Isoimperatorin promotes cell viability in hPDLCs induced by LPS

**Figure 1:**
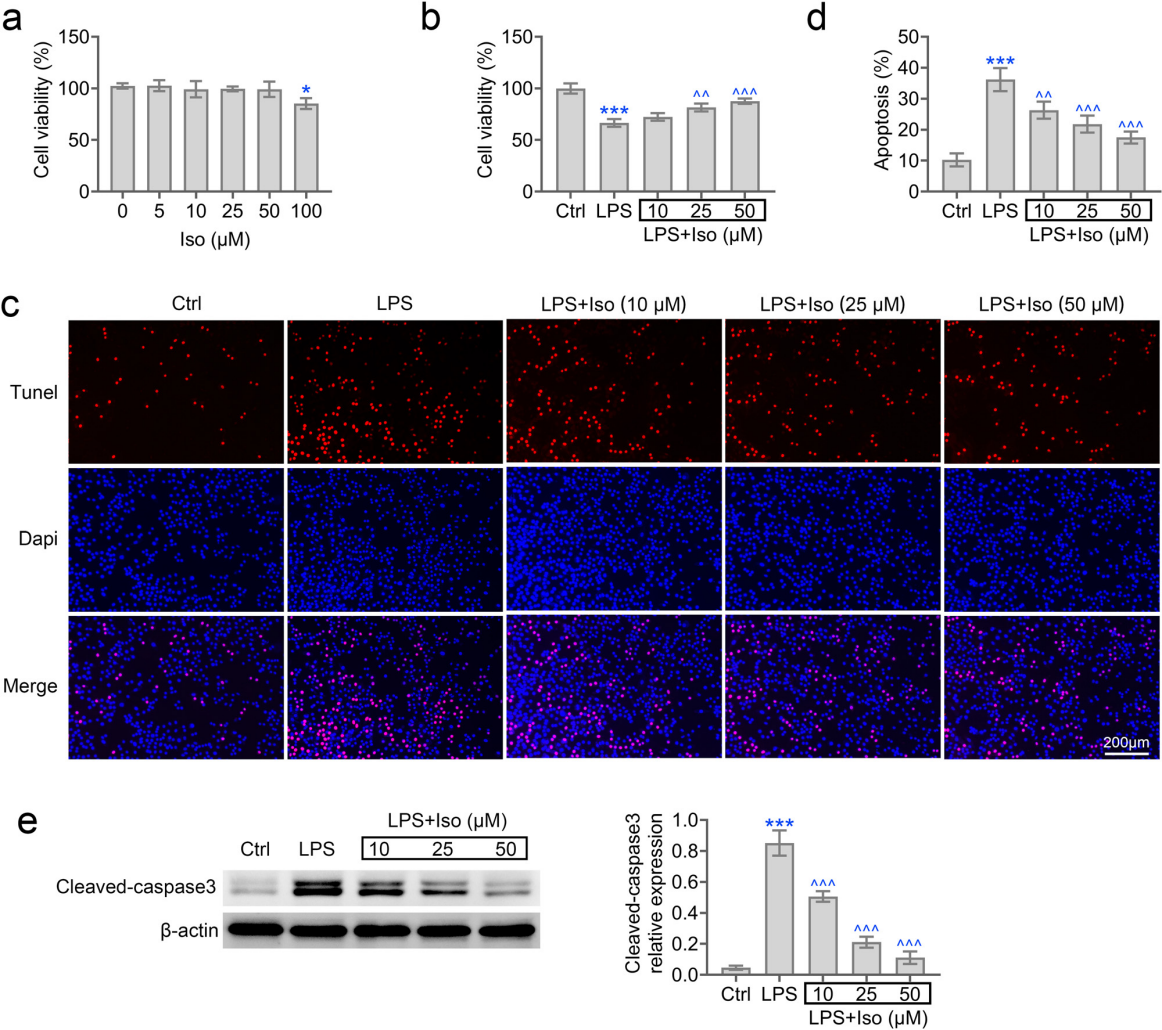
Isoimperatorin promotes cell viability in hPDLCs induced by LPS. (a) The cell viability of hPDLCs in response to increasing isoimperatorin was measured by CCK-8 assay (b) The cell viability of hPDLCs in response to LPS and elevated doses of isoimperatorin were measured by CCK-8 assay. (c and d) hPDLCs cell apoptosis in response to LPS and elevated level of isoimperatorin were detected by TUNEL assay. Magnification, 200×. The quantification was given in (d). (e) hPDLCs cell apoptosis in response to LPS and elevated level of isoimperatorin were also evaluated by Western blot assay. Each experiment was repeated three times. ***p < 0.001 vs control, ^*p* < 0.05, ^^*p* < 0.01, ^^^*p* < 0.001 vs LPS.

To evaluate the cell viability exposed to isoimperatorin in hPDLCs, 3-(4,5-Dimethylthiazol-2-yl)-2,5-diphenyltetrazoliumbromide (MTT) assay was used. hPDLCs were evaluated by isoimperatorin (Figure 1a). Then cells exposed to LPS and isoimperatorin were subjected to the MTT assay. LPS stimulation reduced cell viability in hPDLCs. Isoimperatorin improved cell viability in a dose-dependent manner (Figure 1b). Then, cell apoptosis in isoimperatorin-treated hPDLCs was evaluated by TUNEL staining. LPS treatment induced elevated apoptotic cells, while isoimperatorin treatment decreased cell apoptosis (Figure 1c and d). Collectively, isoimperatorin improves cell viability when stimulated by LPS.

### 3.2 Isoimperatorin improves oxidative stress in hPDLCs induced by LPS

**Figure 2:**
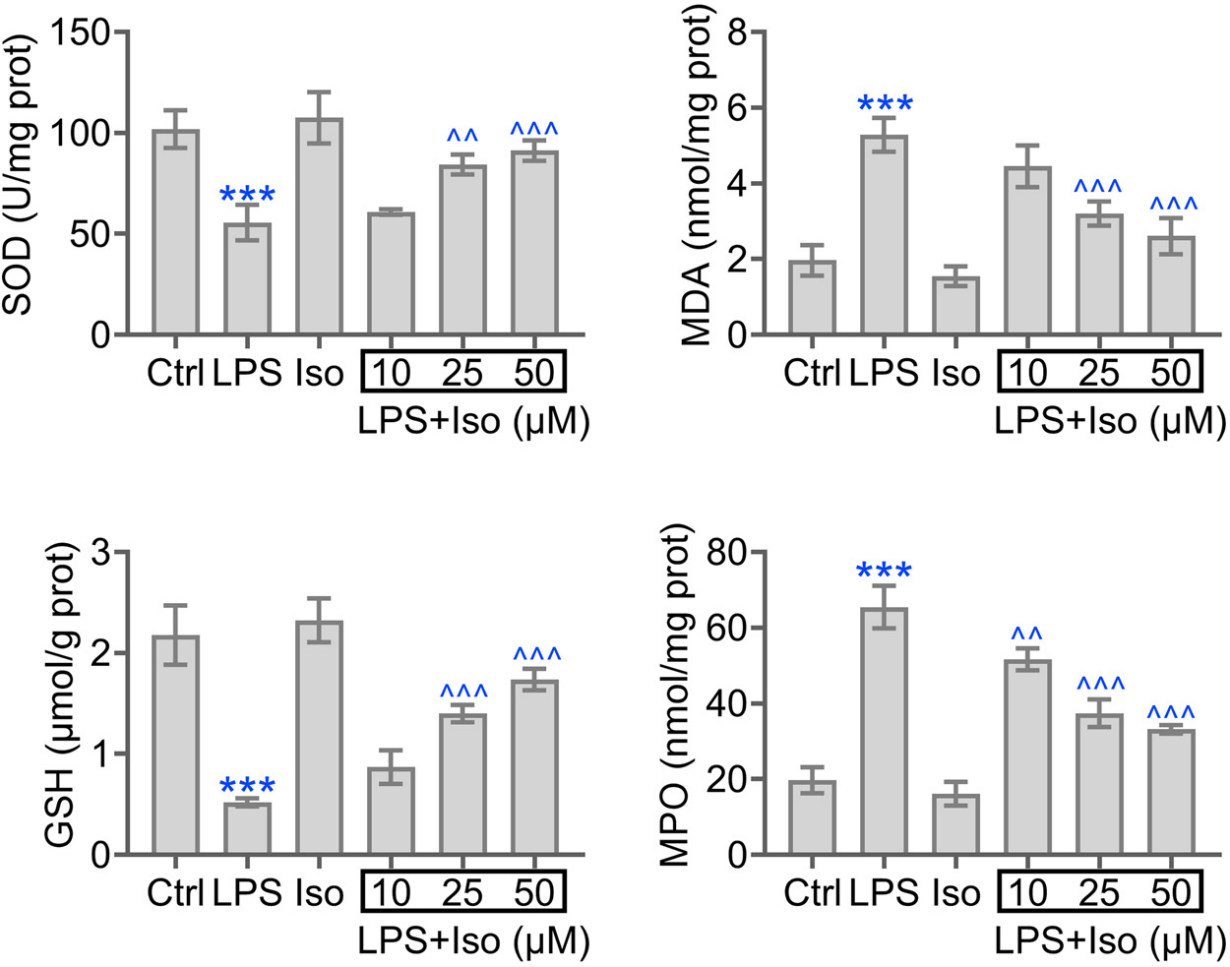
Isoimperatorin relieves oxidative stress in hPDLCs induced by LPS. The level of SOD, MDA, GSH, and MPO in hPDLCs stimulated with LPS and elevated level of isoimperatorin are shown. Each experiment was repeated three times. ***p < 0.001 vs control, ^*p* < 0.05, ^^*p* < 0.01, ^^^*p* < 0.001 vs LPS.

Then we analyzed the level of SOD, MDA, GSH, and MPO in LPS-induced hPDLCs treated with increasing level of isoimperatorin. We observed induction of MDA and MPO and reduction of SOD and GSH in LPS group. Treatment of isoimperatorin reversed the level of SOD, MDA, GSH, and MPO in a dose-dependent manner (Figure 2). In addition, isoimperatorin treatment significantly elevated SOD and GSH levels and reduced MDA and MPO levels compared with the control group (Figure 2). These results suggest that isoimperatorin is associated with reduced oxidative stress in hPDLCs.

### 3.3 Isoimperatorin reduces pro-inflammatory cytokines in hPDLCs

**Figure 3:**
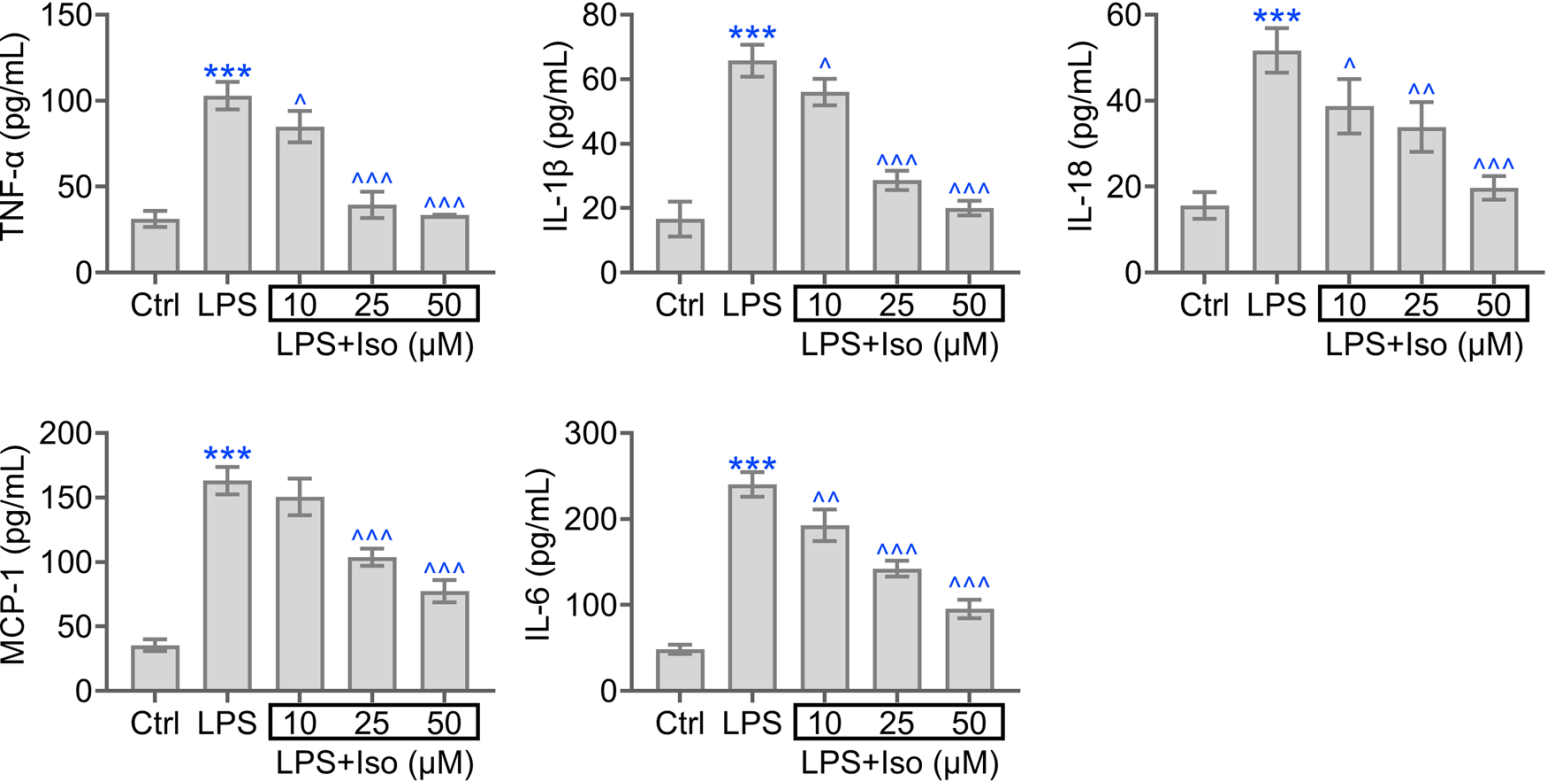
Isoimperatorin inhibits pro-inflammatory cytokines levels in PDLCs. The level of TNF-a, IL-1b, IL-6, MCP-1, and IL-18 in each group was determined. Each experiment was repeated three times. ***p < 0.001 vs control, ^*p* < 0.05, ^^*p* < 0.01, ^^^*p* < 0.001 vs LPS.

The inflammation response in hPDLCs cells was determined by ELISA assay. LPS stimulation enhanced inflammation as revealed by the increased level of TNF-a, IL-1b, MCP-1, IL-6, and IL-18 (Figure 3). Isoimperatorin treatment reduced the level of TNF-a, IL-1b, MCP-1, IL-6, and IL-18 in hPDLCs. Thus, isoimperatorin reduces LPS-induced pro-inflammatory cytokine levels in hPDLCs.

### 3.4 Isoimperatorin suppresses the differentiation into osteoclasts

**Figure 4:**
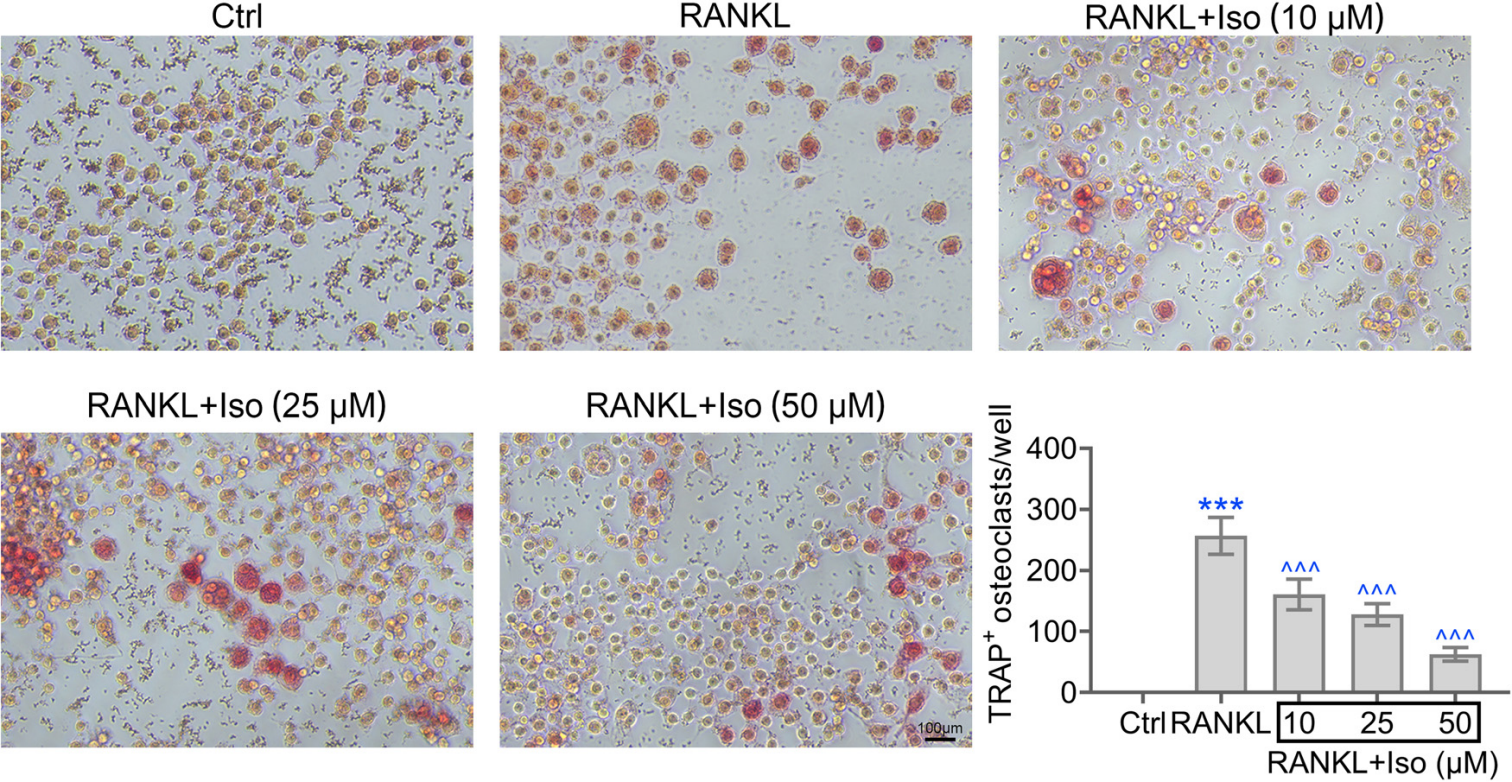
Isoimperatorin inhibits the differentiation of macrophage into osteoclasts. TRAP staining of hPDLCs in response to RANKL and elevated level of isoimperatorin are shown. Each experiment was repeated three times. ***p < 0.001 vs control, ^*p* < 0.05, ^^*p* < 0.01, ^^^*p* < 0.001 vs LPS.

To examine the effects of isoimperatorin on osteoclast differentiation, hPDLCs were induced with RANKL in the presence of isoimperatorin or vehicle. As shown in Figure 4, RANKL significantly induced TRAP^+^-osteoclast differentiation. However, isoimperatorin considerably inhibited the formation of TRAP^+^-osteoclasts. Therefore, isoimperatorin suppresses osteoclast formation.

### 3.5 Isoimperatorin inhibits apoptosis and inflammation of HPDLC by regulating ERK1/2 and NF-κB signaling pathways

**Figure 5:**
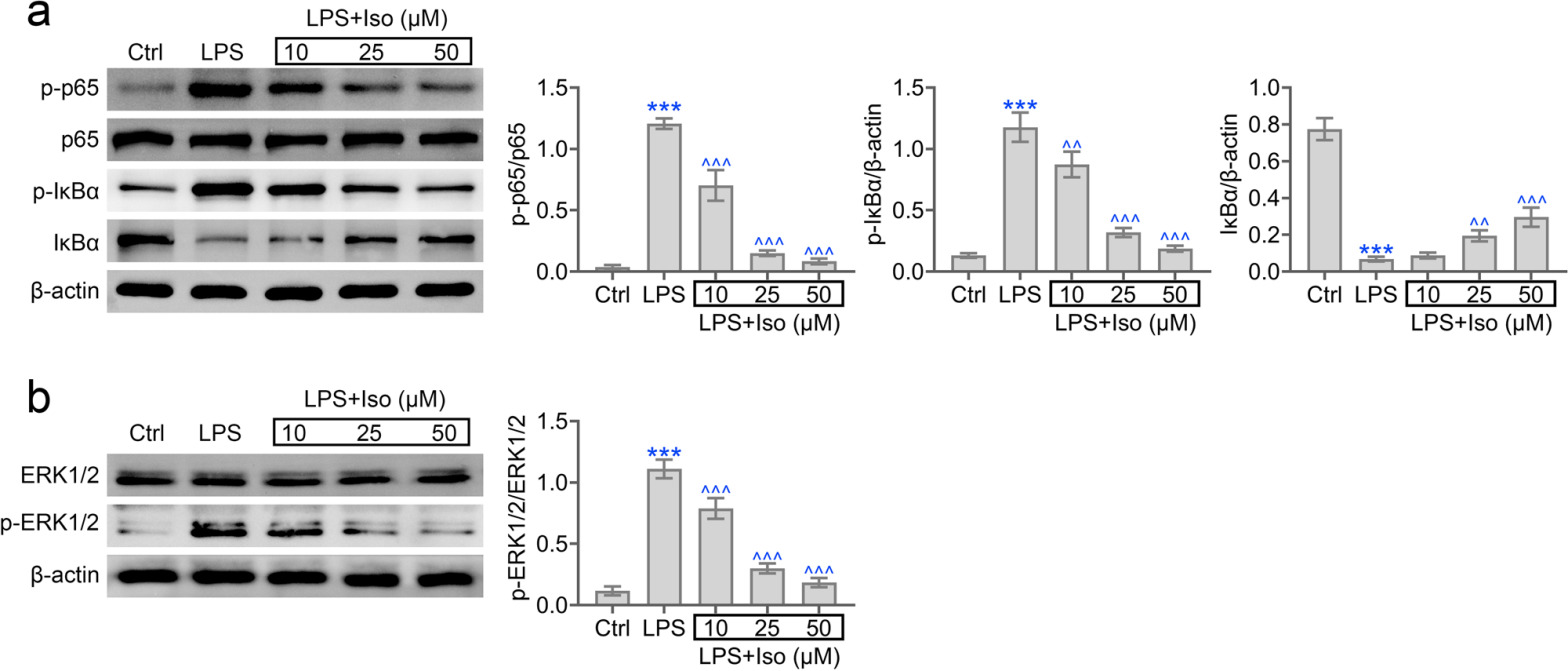
Isoimperatorin regulates ERK and NF-κB signaling pathways in hPDLCs. Immunoblot assays depicted the expression of p-p65, p-IkBα (a) and p-ERK1/2 (b) in LPS and isoimperatorin-induced hPDLCs.

To reveal the involved mechanisms underlying the role of isoimperatorin in periodontitis, the ERK and NF-κB signaling pathways were analyzed. We noticed the elevated level of p-ERK, p-p65, and p-IκBa in LPS-induced hPDLCs (Figure 5). Isoimperatorin inhibited the elevation of these proteins. Our results indicated that isoimperatorin inhibits apoptosis and inflammation of hPDLC by regulating ERK and NF-κB signaling pathways.

## 4 Discussion

Periodontitis is a chronic inflammation of periodontal supporting tissue caused mainly by local factors [14]. If periodontitis is not treated in time, the inflammation may spread from the gingiva deep into the periodontal membrane [15]. Because there is no obvious conscious symptoms at the early stage, it is easy to be ignored. When there are symptoms, it is more serious and even cannot keep teeth. Alveolar bone loss is associated with long-term osteoclast activation [16]. Periodontal pathogens induce periodontal tissue inflammation and immune response, promote the expression of a variety of cytokines, which in turn stimulate alveolar bone absorption, and ultimately lead to tooth loss [17]. Therefore, to treat periodontitis more effectively, it is necessary to conduct in-depth research on its pathogenesis and effectively treat osteoclast activation and inflammatory response, so as to improve the therapeutic effect. In this study, we revealed that isoimperatorin could serve as a promising drug for the periodontitis effect.

LPS stimulation can effectively simulate periodontitis and is a good model for *in vitro* study. Through MTT and TUNEL assays, we revealed that isoimperatorin reduced LPS-induced periodontal membrane cell viability. Through ELISA and immunostaining assays, we revealed that isoimperatorin alleviated the oxidative stress and inflammation response of periodontal membrane cells. Through TRAP and immunoblot, we confirmed isoimperatorin suppressed the osteoblast differentiation. All these findings confirmed that isoimperatorin could serve as a promising drug for periodontitis treatment. In fact, isoimperatorin has anti-inflammatory, analgesic, antitumor, antivirus, and other extensive pharmacological effects [18,19]. It can also regulate PPAR γ and C/EBP α through Akt signaling pathway to promote adipocyte differentiation and prevent diabetes [20]. It can further improve mitochondrial function and protect against acute liver injury caused by carbon tetrachloride. It also activated autophagy by downregulating the mTORC1 signaling pathway and improved cartilage degeneration in osteoarthritis mice [21]. These studies confirmed that isoimperatorin could serve as a drug for multiple types of diseases.

Studies have shown that the pathogenesis of periodontal disease is related to the imbalance of oral resident bacteria and immune and inflammatory responses. Periodontal tissue cells and immune cells secrete a variety of cytokines and effector molecules under inflammation [18,22]. Among them, IL-1, TNF-α, prostaglandin E2, matrix metalloproteinase, and reactive oxygen species (ROS) can not only promote the degradation of connective tissue but also promote the secretion of the RANKL and accelerate osteoclast formation and bone tissue destruction [23,24,25]. Recent studies have confirmed that oxidative stress caused by excessive ROS may be involved in the pathogenesis of chronic periodontitis, and endogenous and exogenous antioxidants have a potential therapeutic value. We found that isoimperatorin alleviated periodontitis via mediating oxidative stress and inflammation response.

In this study, we also revealed that isoimperatorin inhibited the activation of ERK1/2 and NF-κB pathways and therefore alleviated periodontitis. Since the previous study showed that ERK and NF-kapaB pathway could mediate cell antioxidant and inflammatory [26], we thought isoimperatorin acts directly on the reduction of inflammatory factors.

In fact, these pathways are critical in mediating inflammatory and oxidative stress in different diseases [27]. In addition, these pathways also mediated the progression of osteoblast differentiation, which was confirmed by several studies [28]. Therefore, we believed that ERK1/2 and NF-κB pathways could serve as promising targets for periodontitis treatment. On the one hand, isoimperatorin treatment affected cell survival, and on the other hand, it also had a clear effect on cellular oxidative stress through a signaling pathway, ERK1/2 and NF-κB pathway, whose effects on proliferation and oxidative stress have been previously reported.

In conclusion, we revealed that isoimperatorin reduced LPS-induced activity of periodontal cells, alleviated oxidative stress of periodontal cells, reduced secretion of pro-inflammatory factors, and reduced osteoclast formation. Further studies demonstrated that isoimperatorin inhibited LPS-induced ERK1/2 and NF-κB pathways in periodontal cells.
